# Increased physical activity improves gut microbiota composition and reduces short-chain fatty acid concentrations in older adults with insomnia

**DOI:** 10.1038/s41598-022-05099-w

**Published:** 2022-02-10

**Authors:** Faiga Magzal, Tamar Shochat, Iris Haimov, Snait Tamir, Kfir Asraf, Maya Tuchner-Arieli, Carmel Even, Maayan Agmon

**Affiliations:** 1 Laboratory of Human Health and Nutrition Sciences, MIGAL-Galilee Research Institute, Kiryat Shmona, Israel; 2 Nutritional Science Department, Tel Hai College, Upper Galilee, Kiryat Shmona, Israel; 3 The Cheryl Spencer Institute for Nursing Research, University of Haifa, 199 Aba Khoushy Ave. 3498838, Mount Carmel, Haifa, Israel; 4 Department of Psychology, The Max Stern Yezreel Valley College, Jezreel Valley, Israel

**Keywords:** Chemical biology, Computational biology and bioinformatics, Diseases

## Abstract

Physical activity (PA) can improve functional abilities, well-being, and independence in older adults with insomnia. Studies have shown that PA may be linked to changes in the gut microbiota composition and its metabolites’ concentrations. This association among older adults with insomnia, however, is yet to be determined. We explored the relationships between physical activity (PA) levels, gut microbiota composition, and short-chain fatty acid (SCFA) levels in this population. Forty-nine community-dwelling adults with insomnia symptoms, aged 65 and older, participated in this study. Their average daily step-count and sleep continuity measures over a two-week period were calculated based on Actigraphic recordings. Each participant provided fecal samples for the microbiome and SCFA analyses, anthropometric measures, and information via questionnaires on medical history and food consumption. The gut microbiota composition and SCFA concentrations were determined by next-generation sequencing and Gas chromatography-mass spectrometry, respectively. Participants were divided into two groups (more and less active) according to their median step/day count. We compared the microbiota abundance and SCFA concentrations between groups and performed correlation analysis between gut microbiota abundances and study variables. Different microbiota taxa in each PA group and increased SCFAs in feces of less active individuals were found. Changes in step counts were positively or negatively associated with the relative abundance of 19 ASVs, 3 microorganisms at the family level, and 11 microorganisms at the genus level. Furthermore, significant associations were discovered among physical activity, gut microbiota, SCFAs, and sleep parameters. Our findings provide new insights on the relationship between PA, gut microbiota composition, and primary metabolites in older adults with insomnia.

## Introduction

Sleep disturbances are highly prevalent in older adults, with insomnia the most common sleep disorder^1^. Insomnia is defined as a “chronic or acute sleep disorder characterized by a complaint of difficulty initiating, and/or maintaining sleep, and/or a subjective complaint of poor sleep quality that results in daytime impairment and subjective report of impairment”^2^. Its symptoms affect around 50% of the older adult population^3^ and are associated with adverse health outcomes such as systemic inflammation^4^ and all-cause mortality^5^. Numerous studies have explored the link between sleep and physical activity (PA)^6^. Regular PA is thought to be an efficacious, safe, and cost-saving intervention method for those who experience inadequate sleep quantity or quality^6^ and has been shown to improve insomnia and sleep complaints in community-dwelling older adults^7–9^.

To evaluate physical activity in this population, daily step count is an essential as well as easily and securely implemented^10^ tool; the recommended daily step count may vary according to the population characteristics^11^. Although recent studies recommend a dose of 7000–8000 steps per day^11^, a minimum of 6000 daily steps (the upper quartile of community-dwelling older adults) has been shown to improve health (i.e., reduce mortality and frailty)^12,13^. For older adults with insomnia, the accurate dose–response has yet to be determined and should be linked to specific and measurable outcomes such as changes in the gut microbiome composition. Indeed, studies are accumulating at present regarding the link between microbiota profile and its metabolites to PA in older adults^14–17^.

PA and specifically aerobic exercise training have been shown to increase intestinal *Bacteroides* while improving cardiorespiratory fitness in healthy older women^18^. Regular exercise has been shown to reshape aging-induced alterations in microbial composition and function^19^. By contrast, short-term endurance exercise has little effect on gut microbiota composition in older individuals. Five weeks of endurance exercise, for example, caused few changes in older men’s microbiome profile (i.e., increased *Oscillospira* and decreased *Clostridium difficile*)^20^. Furthermore, a combination of moderate resistance training and *Bifidobacterium* spp. supplementation improved cognitive function, body weight, and bowel movement frequency in healthy older adults^21^. Short-chain fatty acids (SCFAs), the main fermentation metabolites of dietary fibers produced by the gut microbiota, were suggested as possible pivots underlying the relationship between PA and health^14^.

In general, SCFAs are linked to improved gut health through several effects, including intestinal barrier integrity^22^, mucus production^23^, and serotonin release, a key regulator of gastrointestinal secretion and motility^24^. Interestingly, higher SCFAs in stool were associated with negative outcomes such as gut microbiome dysbiosis, obesity, hypertension, and cardiometabolic disease risk factors^25^. These contradictory effects may be explained by a lower absorption of metabolites from the human rectum and distal colon since their circulation levels were shown to be inversely associated with those in feces^26^.

In neurobiological diseases such as insomnia, SCFAs have been thought to function as mediators linking gut bacteria to mechanisms in the brain^27,28^. Zhang et al. showed differences in gut microbiota, serum metabolites, and serum immune factors between healthy controls and older adults with insomnia^29^. A recent study revealed a positive association between a more severe insomnia phenotype in older adults and higher SCFA levels in feces^30^. Moreover, severe insomnia was also linked to an increased inflammatory state in the gut^31^. This study examines the effect of physical activity on gut microbiota composition and SCFA concentrations among community-dwelling older adults with insomnia. We also suggest a potential mechanism that explains the contribution of PA to the microbiome profile and SCFA stool levels in this population.

## Methods

The study was carried out in accordance with the Declaration of Helsinki. The institutional review board (IRB) of the Faculty of Social Welfare and Health Sciences at the University of Haifa approved this study and all its methods, conforming to relevant guidelines and regulations (approval number 026/17). All study participants signed informed consent forms. This research received funding from the Israeli Ministry of Science and Technology grant number 3-13607.

### Participants

Sixty-eight community dwelling older adults with insomnia, ages 65 and older^32^, were recruited from community centres in Israel. All participants had no significant visual or hearing impairments, chronic pain, substantial medical, neurological, or psychiatric illness, alcohol or drug abuse, or psychiatric medication. Based on self-report, participants had no sleep apnea syndrome (SAS) or periodic limb movement disorder during sleep (PLMD). No cognitive impairment was reported according to the Mini-Mental State Examination (MMSE < 26)^33^. Insomnia was defined according to Diagnostic and Statistical Manual of Mental Disorders, 5th edition (DSM-5)^2^, and its symptoms were diagnosed from self-report and confirmed by two weeks of actigraphic recordings based on accepted benchmark criteria (sleep onset latency [SOL]) or wake time after sleep onset (WASO) of ≥ 31 min, and < 85% sleep efficiency (SE, percentage of total sleep time after initial sleep onset) for at least three out of seven nights each week). The participants were then divided into two groups according to their median daily step counts: a more active group (step counts ≥ 6500) and a less active group (step counts < 6500). Step-count cutoff was in consonance with previous studies^11–13^ and in line with the recommendations for older adults with various morbidities^11^. Participants were asked to provide a fecal sample and anthropometric measures and to fill in medical history and food consumption questionnaires. Nineteen adults (16 from the less active group and three from the more active group) did not return fecal samples and were excluded from the current analysis. A flowchart summarizing the study stages can be found below.
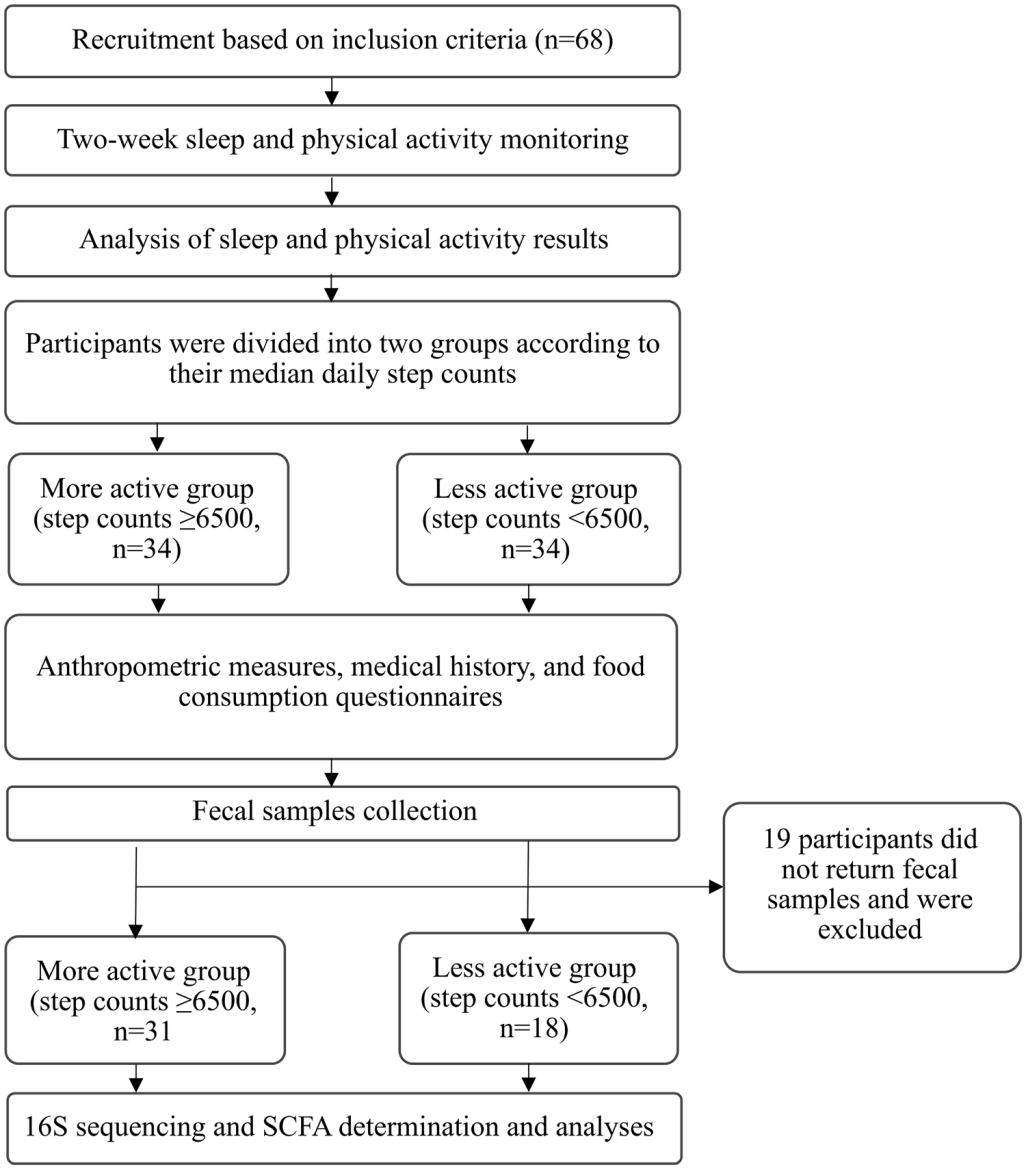


Required sample size was calculated via G*Power (v.3.1)^34^ based on hypothesized group difference in number of steps and was determined to be n = 17 pre group. We assumed a two-tailed t-test, with large effect size for group difference (d = 1), α = 0.05, and 80% power to detect an effect.

### Measures

#### Physical activity and sleep measurements

Physical activity levels, operationalized as an average daily step count, were assessed objectively over two weeks of daily measurement using a GT9X ActiGraph triaxial accelerometer (ActiGraph, Phillips Respironics, Pensacola, FL) worn on the right ankle^35^. Sleep recordings were performed in one-minute epochs with the Actiware 6.0.9 algorithm using the same instrument. Sleep onset and offset were set to the first and last epoch of ten consecutive immobile minutes, respectively. To establish and mark the timing of rest intervals, participants were instructed to first press on the event marker, a built-in feature of the Actiwatch, at bedtime and at final awakening, and then to complete a sleep diary each morning upon awakening. Rest intervals were based primarily on event markers and were verified by sleep diary records. Derived measures included total sleep time (TST, minutes of sleep from intended bedtime to final wake time), sleep onset latency (SOL, minutes to fall asleep from bedtime), sleep efficiency (SE, percentage of total sleep time after initial sleep onset), and wake time after sleep onset (WASO, total wake minutes after sleep onset).

#### Demographic, anthropometric, medical, and nutritional assessment

The participants filled in demographic, anthropometric, and medical history questionnaires. A valid Food Frequency questionnaire was administrated by a registered dietitian and used to evaluate the consumption of macro and micronutrients^36^. Consumption levels of total fibers and proteins were compared between groups.

### Fecal sample analyses

#### Sampling

Participants received clear instructions for the collection of samples at their homes. A fecal sample of each participant was self-collected during the morning hours, stored in screw-capped collection containers filled with an RNase inhibitor solution (DNA/RNA Shield Fecal Collection Tube, Zymo Research, CA, USA), and kept at room temperature for a maximum of one week, transported at room temperature to the laboratory, and kept at − 80 °C until analysis.

#### DNA extraction and 16S rRNA amplification and sequencing (Illumina MiSeq Platform)^37^

DNA was extracted from all fecal samples using the DNeasy PowerSoil Kit (Qiagen, Hilden, Germany), according to the manufacturer’s instructions. Barcoded universal primers 515F (ACACTGACGACATGGTTCTACAGTGCCAGCMGCCGCGGT) and 806R (TACGGTAGCAGAGACTTGGTCTGGACTACHVGGGTWTCT) containing Illumina adapter sequences, which target the highly conserved V4 region, were used to amplify the microbiota from individual samples. Amplification consisted of 20 cycles of 98 °C for 10 s, annealing at 55 °C for 10 s, and 72 °C for 20 s, followed by 1 min at 72 °C. The second PCR was done using the Access Array primers for Illumina (Fluidigm) to add the adaptor and index sequences to the samples for Illumina sequencing.

Amplicons were purified using AMPure XP beads (Beckman Coulter, CA, USA) and subsequently quantified using Qubit (Life Technologies, CA, USA); size was determined by Tapestation (Agilent Technologies, CA, USA). The samples were then loaded on the Miseq (TermoFisher, MA, USA) and sequenced using the Miseq V2 (500 cycles) kit to generate paired-end reads of 2 × 250 bases.

#### Sequencing data analyses

Bacterial sequences were analyzed using the packages “Dada2” and “pyloseq” uploaded to the RStudio software version 4.1.0^38^. Sequences were manually inspected and quality-filtered. A maximum length cut-off was set to 175 bp, discarding forward and reverse reads with an expected error rate higher that 3 nucleotides per 100 bp after truncation. Paired reads were merged, chimeras were removed and an ASVs table was generated.

All further analyses were made with the MicrobiomeAnalyst software platform^39^. Taxonomy was assigned using the Silva reference database version 135.1^40^ and samples with at least 11,500 read counts were included in the analysis (1 sample dropped). Further filters included the removal of low count in samples (10% cut-off for prevalence). The data was normalized by Cumulative Sum Scaling (CSS) except for the alpha diversity analysis.

#### SCFA extraction

SCFA extraction and gas chromatography-mass spectrometry (GC–MS) analysis were performed as described by Magzal et al.^30^. Briefly, samples were thoroughly mixed for 5 min using a vortex. Their pH was then adjusted to 2–3 using orthophosphoric acid (16%v/v) and maintained at room temperature for 10 min. Samples were centrifuged at 4 °C for 5 min at 10,000 rpm, and the supernatant transferred to a vial for GC–MS analyses. 2-methyl-butyric-acid (#109,959, Sigma-Aldrich, MO, USA) was added to each vial as an internal standard at a final concentration of 0.001 M. All vials were stored at -20 °C before GC–MS analyses. The fecal sediment obtained after centrifugation was dried at 60 °C for 5–7 days, and its weight determined.

#### GC–MS analysis

A sample volume of 1μL was injected into a 0.25 mm × 30 m × 0.25 μm fused-silica capillary column with a free fatty acid phase (DB-FFAP 122–3232, Agilent Technologies, CA, USA) of a gas-chromatographer (Agilent 7890A, Agilent Technologies, CA, USA) equipped with an automatic liquid sampler (Gerstel, Mülheim an der Ruhr, Germany). The initial oven temperature was 70 °C, held for 0.75 min, raised to 160 °C at 5 °C/min, raised to 230 °C at 20 °C/min and held for 5.0 min, totaling 27.25 min of runtime. A glass liner with a glass wool plug at its lower end was used to avoid contamination of the GC column with nonvolatile fecal material. Detection was performed by a mass detector coupled to the system (Agilent 5975C) and was operated in the selection ionization mode (SIM). Ion selection of the SCFAs was based on the retention time of standard compounds (WSFA-4, #47056, Sigma-Aldrich, MO, USA). Chemstation software (Hewlett–Packard, CA, USA) was used for acquisition.

### Statistical analyses

Statistical analyses of step numbers, demographics, sleep, dietary and health-related measures between the physical activity groups were performed using SPSS version 26 (IBM SPSS Statistics, New York, US). The comparison of gut microbiota composition between high and low PA groups was performed using the MicrobiomeAnalyst^39^ platform. Parametric data are presented as mean ± standard deviation, and non-parametric data are presented as frequencies (percentage and number of subjects). Demographic, anthropometric, medical, and nutritional data include age, body mass index (BMI), years of education, daily protein and fiber intake, gender, diagnoses of high blood pressure, high cholesterol, diabetes, and heart conditions, as well as uses of anti-cholinergic, depression, and sleep medication. These variables were analyzed via independent sample t-tests, Mann–Whitney U tests, Pearson’s chi-squared tests, or Fisher's exact tests. The outcome measures were the concentration (μmol per gram dry feces) of total SCFAs and of the individual SCFAs: acetate, propionate, isobutyrate, butyrate, isovalerate, and valerate. Since the distribution of each of the SCFAs violated the assumption of normality, the difference between the physical activity groups was analyzed via Mann–Whitney U tests. Data are presented as raw mean ± SD and mean rank, and we report both U and Z statistics. The Monte Carlo sampling method (200,000 samples) was used to estimate exact *p*-values; the reported *p*-values are the Monte Carlo point estimates. The effect size estimator for the significant differences was eta-squared (η^2^). The Shannon index was used to determine Alpha Diversity within samples and the Bray–Curtis index facilitated the analysis of phylogenetic differences between both groups (β-diversity). The algorithm metagenomesSeq (zero-inflated Gassian fit) enabled identification of microorganisms that are differently abundant between both groups. The Spearman correlation analysis were performed using the package “metagenomeseq”, RStudio software (version 4.1.0)^38^. The data was normalized by Cumulative Sum Scaling (CSS). Only ASVs or taxa with prevalence of above 10% were considered for analysis (i.e., > 5 samples).

## Results

### Descriptive statistics

The median daily step counts for the less active and the more active group were significantly different (5544.90, IQR = 4269.04–6239.50 and 8367.80, IQR = 7555.00–10,505.16, respectively) (Table 1). Comparison analysis of demographic, dietary and health-related variables indicated no differences between both groups in age, BMI, years of education, gender, and daily protein and fiber intake. The groups did not differ significantly in the percentage of participants diagnosed with high blood pressure, high cholesterol levels, diabetes, a heart condition, or who used anti-cholinergic, depression, and sleep medication. Likewise, no significant differences in sleep parameters were detected between groups (Table 1).

**Table 1:** Comparisons of step counts, demographic, sleep, and dietary and health-related measures between the physical activity groups.

Parameter	Lower physical activity (less active) (n = 18)	Higher physical activity (more active) (n = 31)	Statistics	*P*	η^2^
Number of steps	Median = 5544.90 IQR = 4269.04–6239.50	Median = 8367.80 IQR = 7555.00–10,505.16	U = 558 (Z = 5.78)	** < 0.001**	0.683
**Demographic measures**					
Age	73.66 ± 6.65	72.22 ± 5.08	*t*_(47)_ = 0.85	0.398	
BMI (kg/m^2^)	27.756 ± 3.27	25.81 ± 3.94	*t*_(47)_ = 1.76	0.084	
Years of education	15.44 ± 2.50	16.45 ± 2.24	*t*_(47)_ = − 1.45	0.153	
% of Females	77.77 (n = 14)	80.64 (n = 25)	^	> 0.999	
**Sleep measures**					
Sleep time (Hours)	24.04 ± 0.87	24.08 ± 1.14	*t*_(47)_ = − 0.12	0.900	
Wake time (Hours)	7.17 ± 1.02	6.79 ± 1.04	*t*_(47)_ = 1.23	0.221	
Total sleep time (TST) (minutes)	427.69 ± 37.78	403.30 ± 63.63	*t*_(46.94)_ = 1.68	0.098	
Sleep onset latency (SOL) (minutes)	Median = 15 IQR = 7.35–26.55	Median = 11.78 IQR = 6.78–28.40	*U* = 259.5 (*Z* = − 0.40)	0.685	
Sleep efficiency (SE) (%)	Median = 81.95 IQR = 76.85–85.21	Median = 83.21 IQR = 79.22–86.45	U = 314 (Z = 0.72)	0.467	
WASO (minutes)	Median = 55.46 IQR = 48.85–71.52	Median = 47.14 IQR = 32.44–60.00	U = 204 (Z = − 1.55)	0.119	
**Dietary and health-related measures**					
Protein intake (g/day)	Median = 70.40 IQR = 58.58–108.86	Median = 97.56 IQR = 77.41–126.36	*U* = 369.50 (*Z* = 1.89)	0.058	
Fiber intake (g/day)	Median = 28.77 IQR = 23.95–39.65	Median = 31.82 IQR = 27.32–47.43	*U* = 327 (*Z* = 1.00)	0.316	
High blood pressure (%)	55.55 (n = 10)	41.93 (n = 13)	***χ***^***2***^_(1)_ = 0.84	0.357	
High cholesterol (%)	38.88 (n = 7)	48.38 (n = 15)	***χ***^***2***^_(1)_ = 0.41	0.519	
Diabetes (%)	22.22 (n = 4)	25.80 (n = 8)	^	> 0.999	
Heart condition (%)	16.66 (n = 3)	12.90 (n = 4)	^	0.697	
Depression medication (%)	5.55 (n = 1)	16.12 (n = 5)	^	0.393	
Sleep medication (%)	11.11 (n = 2)	12.90 (n = 4)	^	> 0.999	
Anti-cholinergic medication (%)	16.66 (n = 3)	16.12 (n = 5)	^	> 0.999	

*BMI* body mass index, *IQR* interquartile range, *η2* Eta-squared (reported only for significant comparisons), *χ*^*2*^ Chi-square.

^Based on Fisher's exact test Eta-squared (η^2^) is reported only for significant comparisons.

Bold values denote statistical significance at the p < 0.05 level.

### Comparison of gut microbiota composition between high and low PA groups

**Figure 1:**
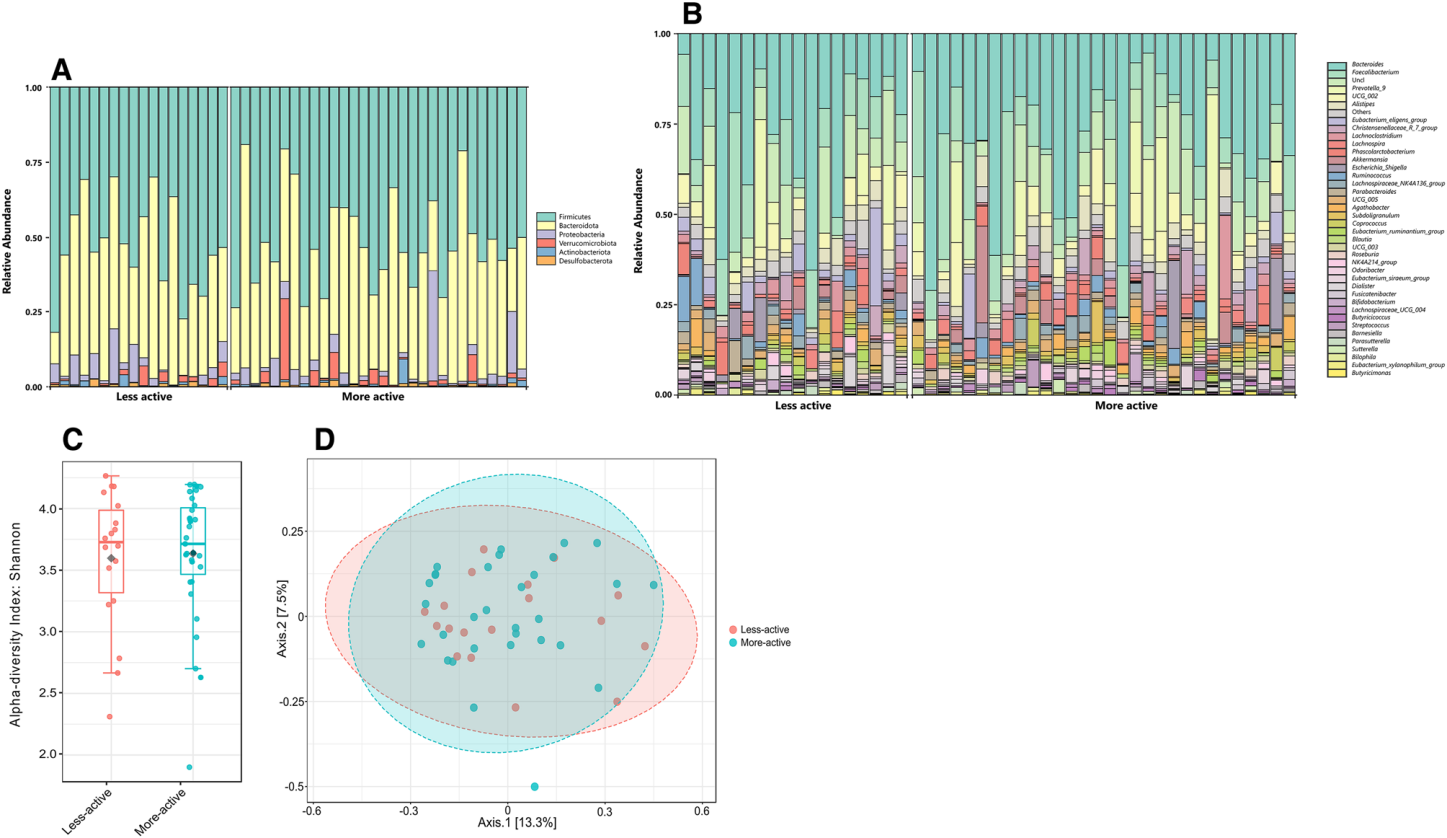
(**A**,**B**) Taxonomic composition distribution in samples at the phylum (**A**) and genus (**B**) levels. The 49 samples are divided into two groups, less active and more active individuals. The bacteria relative abundances are displayed in the histogram in the corresponding proportion and color according to the legend. (**C**,**D**) Boxplots showing alpha diversity the feature level (**C**) (Shannon index) and beta diversity at the feature level (**D**) (Bray–Curtis index). Plots in blue represent the data from the more-active group, plots in red are from the less-active group.

We evaluated the composition of all participants’ gut microbiota at the phylum and genus levels (Fig. 1A,B). The most abundant phyla were Firmicutes (52.14%), Bacteroidota (39.65%), followed by *Proteobacteria* (4.99%), Verrucomicrobia (1.98%), Actinobacteriota (0.71%) and Desulfobacterota (0.52%). At the genus level, the 6 most abundant gut microbiota were *Bacteroides* (27.94%), *Faecalibacterium* (11.61%), *Prevotella 9* (5.67%) followed by *UCG 002* (4.69%), *Alistipes* (3.30%) and *Eubacterium eligens group* (2.52%).

Based on relative abundance, higher step counts did not cause changes in microbiota diversity within individuals (Shannon Index, Mann–Whitney, p = 0.493) and in microbiota divergence between individuals (Beta diversity, PERMANOVA, p < 0.394) (Fig. 1C,D).

We performed a screening of the gut microbiota at ASVs and family and genus levels between more active and less active groups using the metagenomeseq method. More active individuals had significantly higher abundances of 13 ASVs, 3 bacteria at the family level and 4 bacteria at the genus level. Seven ASVs were significant higher in less active individuals. The results are summarized in Table 2. *Bifidobacterium, Clostridium *sensu stricto* 1, UCG-002, Catenibacterium, Peptococcus, Holdemanella and Butyricicoccus* are among the genera present in more active individuals. Less active ones show a higher relative abundance of the genera *Barnesiella, Blautia, Lachnoclostridium, Christensenellaceae R-7 group* and *UCG-005.*

**Table 2:** Significant microbiota ASVs, families and genera present in the more active and less active groups.

ASVs	Phylum	Class	Order	Family	Genus	Species	More active		Less active		Physical activity	
							Average	stdev	Average	stdev	p value	FDR
**ASVs present in the more**												
91	*Actinobacteriota*	*Actinobacteria*	*Bifidobacteriales*	*Bifidobacteriaceae*	*Bifidobacterium*		3.091	0.094	0.232	0.007	1.92E−03	*0.031*
22	*Bacteroidota*	*Bacteroidia*	*Bacteroidales*	*Bacteroidaceae*	*Bacteroides*	*plebeius*	3.081	0.078	0.249	0.006	5.18E−03	*0.045*
184	*Firmicutes*	*Bacilli*	*Erysipelotrichales*	*Erysipelatoclostridiaceae*	*Catenibacterium*		3.226	0.089	0.000	0.000	1.81E−09	*2.02E*−*07*
212	*Firmicutes*	*Clostridia*	*Clostridia UCG-014*				2.849	0.048	0.649	0.024	4.54E−06	*3.39E*−*04*
642	*Firmicutes*	*Clostridia*	*Clostridia vadinBB60 group*				2.989	0.054	0.407	0.013	2.61E−05	*1.46E*−*03*
517	*Firmicutes*	*Clostridia*	*Clostridiales*	*Clostridiaceae*	*Clostridium *sensu stricto* 1*		2.751	0.057	0.818	0.029	5.25E−05	*1.68E*−*03*
444	*Firmicutes*	*Clostridia*	*Lachnospirales*	*Lachnospiraceae*	*[Ruminococcus] gauvreauii group*		2.596	0.037	1.085	0.032	3.06E−03	*0.043*
524	*Firmicutes*	*Clostridia*	*Lachnospirales*	*Lachnospiraceae*			2.442	0.043	1.350	0.034	4.72E−03	*0.044*
555	*Firmicutes*	*Clostridia*	*Oscillospirales*	*Butyricicoccaceae*			2.350	0.066	1.509	0.048	4.49E−04	*0.008*
63	*Firmicutes*	*Clostridia*	*Oscillospirales*	*Oscillospiraceae*	*UCG-002*						9.55E−14	*2.14E*−*11*
120	*Firmicutes*	*Clostridia*	*Oscillospirales*	*Oscillospiraceae*			2.706	0.037	0.896	0.014	4.40E−03	*0.044*
101	*Firmicutes*	*Clostridia*	*Oscillospirales*	*Ruminococcaceae*	*[Eubacterium] siraeum group*		3.108	0.090	0.203	0.007	2.00E−04	*4.07E*−*03*
548	*Firmicutes*	*Clostridia*	*Peptococcales*	*Peptococcaceae*	*Peptococcus*		2.891	0.067	0.576	0.013	4.24E−03	*0.044*
**Family level**												
	*Firmicutes*	*Clostridia*	*Peptococcales*	*Peptococcaceae*			0.389	0.314	0.097	0.059	6.01E−06	*2.16E*−*04*
	*Firmicutes*	*Bacilli*	*Erysipelotrichales*	*Erysipelatoclostridiaceae*			5.621	0.121	0.059	0.042	1.39E−04	*2.51E*−*03*
	*Firmicutes*	*Bacilli*	*Erysipelotrichales*	*Erysipelotrichaceae*			0.311	0.276	0.045	0.036	8.03E−04	*9.63E*−*03*
**Genus Level**												
	*Firmicutes*	*Bacilli*	*Erysipelotrichales*	*Erysipelatoclostridiaceae*	*Catenibacterium*		3.258	0.040	0.000	0.000	6.47E−11	*4.85E*−*09*
	*Firmicutes*	*Clostridia*	*Peptococcales*	*Peptococcaceae*	*Peptococcus*		2.995	0.076	0.786	0.186	3.61E−05	*1.36E*−*03*
	*Firmicutes*	*Bacilli*	*Erysipelotrichales*	*Erysipelotrichaceae*	*Holdemanella*		5.134	0.079	2.380	0.002	9.59E−05	*2.40E*−*03*
	*Firmicutes*	*Clostridia*	*Oscillospirales*	*Butyricicoccaceae*	*Butyricicoccus*		8.007	0.080	3.098	0.085	2.59E−03	*0.049*
**ASVs present in the less-active group**												
159	*Bacteroidota*	*Bacteroidia*	*Bacteroidales*	*Barnesiellaceae*	*Barnesiella*		1.092	0.033	3.674	0.068	4.41E−05	*1.65E*−*03*
191	*Firmicutes*	*Bacilli*	*Izemoplasmatales*				0.481	0.014	2.946	0.085	1.24E−04	*0.003*
466	*Firmicutes*	*Clostridia*	*Christensenellales*	*Christensenellaceae*	*Christensenellaceae R-7 group*		1.075	0.039	3.704	0.077	3.44E−03	*0.043*
370	*Firmicutes*	*Clostridia*	*Lachnospirales*	*Lachnospiraceae*	*Blautia*		1.025	0.026	3.790	0.066	3.35E−03	*0.043*
207	*Firmicutes*	*Clostridia*	*Lachnospirales*	*Lachnospiraceae*	*Lachnoclostridium*		1.572	0.052	2.848	0.084	4.96E−03	*0.044*
169	*Firmicutes*	*Clostridia*	*Oscillospirales*	*[Eubacterium] coprostanoligenes group*			1.336	0.023	3.255	0.052	6.05E−03	*0.048*
242	*Firmicutes*	*Clostridia*	*Oscillospirales*	*Oscillospiraceae*	*UCG-005*		1.202	0.027	3.485	0.047	2.62E−03	*0.039*
546	*Firmicutes*	*Clostridia*	*Oscillospirales*	*Ruminococcaceae*			1.735	0.037	2.567	0.061	6.00E−03	*0.048*

*ASVs* amplicon sequence variant, *stdev* standard deviation, *FDR* false discovery rate.

FDR values are shown in italics.

### Comparison of SCFA concentrations between more and less active groups

We determined SCFA concentrations for both groups using GC–MS. Acetate was the most prevalent SCFA in both groups, followed by butyrate and propionate. Analysis of the difference in SCFAs (acetate, propionate, isobutyrate, butyrate, isovalerate, valerate, and total SCFAs) between the groups is presented in Table 3.

**Table 3:** Comparisons of SCFAs between the physical activity groups.

SCFAs (μmol per gram dry feces)	Lower physical activity (less active) (n = 18)	Higher physical activity (more active) (n = 31)	U statistic (Z)	*p*	η^2^
Acetate	149.31 ± 52.81 (Med = 140.68, IQR = 111.76–179.72)	129.44 ± 55.42 (Med = 118.60, IQR = 83.23–167.61)	216 (− 1.3)	0.196	
Propionate	26.7 ± 14.48 (Med = 26.78, IQR = 12.94–41.50)	14.37 ± 6.79 (Med = 13.29, IQR = 9.51 – 18.45)	124.5 (− 2.74)	**0.005**	0.16
Isobutyrate	0.96 ± 1.63 (Med = 0, IQR = 0–1.76)	0.09 ± 0.33 (Med = 0, IQR = 0–02)	164 (− 1.99)	**0.04**	0.05
Butyrate	41.94 ± 28.44 (Med = 34.21, IQR = 19.50–55.44)	31.2 ± 20.71 (Med = 24.31, IQR = 15.42–41.42)	211 (− 1.41)	0.162	
Isovalerate	2.24 ± 1.87 (Med = 1.50, IQR = 0.84–4.05)	1.50 ± 1.43 (Med = 1.23, IQR = 0.35–2.08)	212.5 (− 1.38)	0.172	
Valerate	3.98 ± 2.99 (Med = 3.10, IQR = 1.45–5.53)	1.94 ± 1.47 (Med = 1.59, IQR = 0.86–2.64)	147.5 (− 2.48)	**0.012**	0.13
Total SCFAs	226.34 ± 95.31 (Med = 213.02, IQR = 147.52–308.23)	170.35 ± 69.57 (Med = 156.16, IQR = 119.82–214.50)	171 (− 1.97)	**0.049**	0.08

Data are presented as mean ± SD (median, IQR).

Eta-squared (η^2^) is reported only for significant comparisons.

*SCFA* short-chain fatty acids, *Med* median, *IQR* interquartile range, *η2* Eta-squared.

Bold values denote statistical significance at the p < 0.05 level.

The analysis revealed that the less active group had significantly higher concentrations of propionate, isobutyrate, and valerate compared to the more active group. The magnitude of the difference in concentration between the study groups was high for propionate (η^2^ = 16). The rest of the comparisons had a medium effect size. The less active group also had significantly higher concentrations of total fecal SCFAs, compared to the more active activity group, with a medium effect size (η^2^ = 08).

### Correlations between the gut microbiome and physical activity

To further explore the relationship between microbial changes and physical activity, we performed correlation analyses of all the microbial flora and step counts. Changes in step counts were positively or negatively associated with the relative abundance of 19 ASVs, 3 microorganisms at the family level, and 11 microorganisms at the genus level. In addition, we performed the same correlation analysis of the gut microbiota with the parameters age, BMI, SCFAs, sleep efficiency, and the nutrients protein and fiber. Significant associations are shown in Table 4 and a complete table with all correlations tested can be found in the supplementary Table S1. The family Monoglobaceae and its genus *Monoglobus* were negatively correlated to step counts (p = 0.031) and SOL (p = 0.049) and positively associated with isobutyrate concentrations (p = 0.039) and sleep efficiency (p = 0.022). The genus *Ruminococcus* was negatively correlated to physical activity (p = 0.039) and fiber intake (p = 0.002) but positively associated with isovalerate (p = 0.012). *Anaerovoracaceae FamilyXIII UCG-001* was positively associated with PA (p = 0.021) but negatively associated with propionate (p = 0.036). Positive correlations were found between the genus *UCG-002,* PA (p = 0.009), and protein intake (p = 0.037). The genus *Lachnoclostridium* was negatively correlated with PA (p = 0.017) and protein intake (p = 0.020). The genus *Sutterella* was positively associated with PA (p = 0.047) and negatively correlated to the SCFAs propionate (p = 0.028), and isobutyrate (p = 0.032). *Bacteroides ovatus, Bacteroides clarus, and Bacteroides uniformis* were negatively correlated with PA (p = 0.000, p = 0.010, and p = 0.044, respectively). *Bacteroides putredinis and Blautia hansenii* correlated oppositely with PA (S.corr = -0.333, p = 0.021 and S.corr = 0.332, p = 0.047, respectively) and BMI (S.corr = 0.0331, p = 0.020 and S.corr = -0.249, p = 0.039, respectively).

**Table 4:** Significant Spearman positive and negative correlations among the gut microbiome, physical activity (step counts), and measured parameters.

ASVs		PA		BMI		Propionate		Isobutyrate		Isovalerate		SE		SOL		ET		Protein		Fiber	
		S corr	p value	S corr	p value	S corr	p value	S corr	p value	S corr	p value	S corr	p value	S corr	p value	S corr	p value	S corr	p value	S corr	p value
009	Bacteroidota; Bacteroidia; Bacteroidales; Bacteroidaceae; *B. uniformis*	− 0.289	**0.044**	0.139	0.340	0.018	0.906	0.237	0.634	0.062	0.671	0.001	0.994	− 0.036	0.805	0.079	0.589	0.197	0.174	0.041	0.778
035	Bacteroidota; Bacteroidia; Bacteroidales; Bacteroidaceae; *Bacteroides*	0.294	**0.040**	− 0.021	0.884	− 0.135	0.377	− 0.034	0.968	− 0.094	0.522	− 0.100	0.494	− 0.009	0.949	0.062	0.672	0.224	0.121	0.146	0.318
107	Bacteroidota; Bacteroidia; Bacteroidales; Bacteroidaceae; *Bacteroides; clarus*	− 0.366	**0.010**	0.038	0.796	− 0.116	0.447	− 0.073	0.648	− 0.140	0.337	0.069	0.637	− 0.130	0.372	0.038	0.797	− 0.209	0.149	− 0.119	0.414
049	Bacteroidota; Bacteroidia; Bacteroidales; Bacteroidaceae; *Bacteroides; ovatus*	− 0.483	**0.000**	0.033	0.824	0.173	0.257	0.000	0.306	0.039	0.792	0.101	0.490	− 0.024	0.870	− 0.018	0.903	− 0.242	0.094	− 0.053	0.719
019	Bacteroidota; Bacteroidia; Bacteroidales; Rikenellaceae; *Alistipes; putredinis*	− 0.333	**0.019**	0.331	0.020	0.070	0.647	0.151	0.319	0.025	0.863	− 0.107	0.465	− 0.048	0.741	− 0.025	0.865	− 0.028	0.848	− 0.167	0.252
184	Firmicutes; Bacilli; Erysipelotrichales; Erysipelatoclostridiaceae; *Catenibacterium*	0.367	**0.010**	− 0.241	0.095	− 0.259	0.086	− 0.160	0.327	− 0.196	0.177	− 0.066	0.654	− 0.108	0.458	− 0.172	0.238	0.234	0.105	− 0.041	0.782
212	Firmicutes; Clostridia; Clostridia UCG− 014	0.346	**0.015**	0.017	0.906	− 0.133	0.385	− 0.184	0.252	− 0.002	0.991	− 0.129	0.379	0.148	0.310	− 0.053	0.716	0.127	0.383	− 0.195	0.179
079	Firmicutes; Clostridia; Lachnospirales; Lachnospiraceae; *Lachnospira*	0.339	**0.017**	− 0.034	0.817	− 0.277	0.066	− 0.363	0.723	− 0.061	0.677	− 0.215	0.138	0.211	0.146	0.087	0.553	0.024	0.872	− 0.069	0.636
207	Firmicutes; Clostridia; Lachnospirales; Lachnospiraceae; Lachnoclostridium	− 0.338	**0.017**	0.054	0.710	0.085	0.578	0.152	0.902	− 0.112	0.444	0.008	0.956	0.065	0.659	0.202	0.163	− 0.332	**0.020**	− 0.041	0.778
063	Firmicutes; Clostridia; Oscillospirales; Oscillospiraceae; *UCG-002*	0.367	**0.009**	− 0.209	0.149	− 0.253	0.093	− 0.248	0.951	0.086	0.556	0.015	0.921	− 0.185	0.204	− 0.162	0.266	0.299	**0.037**	0.093	0.525
1018	Firmicutes; Clostridia; Peptostreptococcales-Tissierellales; Anaerovoracaceae; *Family XIII UCG-001*	0.330	**0.021**	− 0.224	0.121	− 0.313	**0.036**	0.075	0.660	− 0.058	0.692	0.033	0.824	− 0.148	0.310	0.092	0.527	0.000	0.998	0.115	0.430
517	Firmicutes; Clostridia; Clostridiales; Clostridiaceae; *Clostridium *sensu stricto* 1*	0.352	**0.013**	− 0.020	0.890	0.156	0.307	− 0.033	0.710	− 0.170	0.242	− 0.041	0.778	0.021	0.887	0.222	0.126	0.060	0.685	0.258	0.074
642	Firmicutes; Clostridia; Clostridia vadinBB60 group	0.349	**0.014**	− 0.146	0.318	− 0.208	0.170	0.002	0.426	− 0.104	0.478	0.133	0.361	− 0.163	0.263	0.115	0.432	0.196	0.178	0.102	0.487
500	Firmicutes; Clostridia; Lachnospirales; Lachnospiraceae	− 0.361	**0.011**	0.086	0.556	0.076	0.622	− 0.012	0.606	− 0.107	0.463	0.047	0.747	− 0.058	0.695	0.053	0.719	− 0.168	0.249	0.057	0.695
494	Firmicutes; Clostridia; Lachnospirales; Lachnospiraceae; *Blautia; hansenii*	0.332	**0.020**	− 0.297	0.039	− 0.222	0.142	− 0.077	0.896	− 0.023	0.876	0.086	0.559	− 0.073	0.617	− 0.091	0.534	0.136	0.351	0.012	0.934
286	Firmicutes; Clostridia; Oscillospirales; [Eubacterium] coprostanoligenes group	0.290	**0.043**	− 0.214	0.139	0.045	0.767	− 0.017	0.592	0.064	0.660	− 0.154	0.290	0.035	0.809	− 0.110	0.452	0.077	0.600	0.054	0.710
353	Firmicutes; Clostridia; Oscillospirales; Ruminococcaceae; *Negativibacillus*	− 0.320	**0.025**	0.083	0.572	0.219	0.149	0.017	0.339	0.180	0.216	0.071	0.629	0.131	0.370	− 0.046	0.755	− 0.063	0.665	− 0.125	0.391
232	Firmicutes; Negativicutes; Veillonellales-Selenomonadales; Veillonellaceae; *Dialister*	0.340	**0.017**	0.062	0.672	− 0.030	0.847	− 0.169	0.159	0.210	0.147	0.142	0.331	− 0.170	0.244	− 0.270	0.061	0.047	0.750	− 0.128	0.382
148	Proteobacteria; Gammaproteobacteria; Burkholderiales; Sutterellaceae; *Sutterella*	0.285	**0.047**	− 0.249	0.085	− 0.328	**0.028**	− 0.043	**0.032**	− 0.274	0.057	0.054	0.714	− 0.024	0.870	− 0.132	0.365	0.017	0.906	0.120	0.411
**Family level**																					
	Bacteroidota; Bacteroidia; Bacteroidales; Marinifilaceae	− 0.287	**0.045**	0.044	0.764	− 0.111	0.468	0.201	0.191	− 0.055	0.709	− 0.037	0.799	0.017	0.905	0.063	0.669	− 0.029	0.845	0.109	0.456
	Firmicutes; Clostridia; Monoglobales; Monoglobaceae	− 0.309	**0.031**	− 0.161	0.270	0.029	0.849	0.312	**0.039**	− 0.034	0.819	0.326	**0.022**	− 0.283	**0.049**	0.141	0.334	− 0.276	0.055	− 0.137	0.346
	Proteobacteria; Gammaproteobacteria; Pseudomonadales; SAR86 clade	0.283	**0.048**	− 0.122	0.405	− 0.097	0.525	− 0.023	0.880	− 0.182	0.210	− 0.222	0.125	0.090	0.539	− 0.086	0.558	0.045	0.756	0.176	0.225
**Genus level**																					
	Bacteroidota; Bacteroidia; Bacteroidales; Rikenellaceae; *Alistipes*	− 0.299	**0.037**	0.082	0.578	0.045	0.771	0.218	0.155	0.134	0.360	− 0.015	0.921	− 0.040	0.786	0.173	0.235	− 0.053	0.716	− 0.033	0.824
	Bacteroidota; Bacteroidia; Bacteroidales; Marinifilaceae; *Butyricimonas*	− 0.305	**0.033**	0.109	0.458	0.102	0.506	0.138	0.371	− 0.023	0.875	0.007	0.963	− 0.084	0.565	0.185	0.203	− 0.029	0.844	− 0.052	0.722
	Bacteroidota; Bacteroidia; Flavobacteriales; Flavobacteriaceae; *NS4 marine group*	0.288	**0.045**	− 0.323	0.023	− 0.118	0.439	0.095	0.542	− 0.134	0.359	− 0.143	0.328	0.003	0.983	− 0.120	0.411	0.122	0.404	0.088	0.549
	Bacteroidota; Bacteroidia; Flavobacteriales; Flavobacteriaceae; *Winogradskyella*	0.291	**0.043**	− 0.071	0.627	0.015	0.921	− 0.117	0.449	− 0.235	0.103	− 0.188	0.195	0.220	0.129	− 0.028	0.850	0.071	0.629	0.105	0.473
	Firmicutes; Bacilli; Erysipelotrichales; Erysipelatoclostridiaceae; *Catenibacterium*	0.367	**0.010**	− 0.241	0.095	− 0.259	0.086	− 0.160	0.301	− 0.196	0.177	− 0.066	0.654	− 0.108	0.458	− 0.172	0.238	0.234	0.105	− 0.041	0.782
	Firmicutes; Clostridia; Monoglobales; Monoglobaceae; *Monoglobus*	− 0.309	**0.031**	− 0.161	0.270	0.029	0.849	0.312	**0.039**	− 0.034	0.819	0.326	**0.022**	− 0.283	**0.049**	0.141	0.334	− 0.276	0.055	− 0.137	0.346
	Firmicutes; Clostridia; Oscillospirales; Ruminococcaceae; *DTU089*	− 0.292	**0.041**	0.184	0.207	− 0.093	0.545	− 0.040	0.795	− 0.013	0.930	0.011	0.941	0.050	0.731	0.029	0.841	− 0.122	0.402	− 0.023	0.877
	Firmicutes; Clostridia; Oscillospirales; Ruminococcaceae; *Negativibacillus*	− 0.428	**0.002**	0.188	0.196	0.269	0.074	0.031	0.843	0.212	0.145	0.089	0.543	0.101	0.491	− 0.101	0.492	0.166	0.255	− 0.196	0.177
	Firmicutes; Clostridia; Oscillospirales; Ruminococcaceae; *Ruminococcus*	− 0.297	**0.039**	0.276	0.055	− 0.064	0.674	0.289	0.057	0.355	**0.012**	− 0.021	0.887	0.013	0.928	− 0.206	0.156	− 0.152	0.297	− 0.429	**0.002**
	Proteobacteria; Alphaproteobacteria; Rhodobacterales; Rhodobacteraceae; *Epibacterium*	0.293	**0.041**	− 0.060	0.682	0.018	0.906	− 0.117	0.449	− 0.239	0.098	− 0.191	0.189	0.225	0.120	− 0.016	0.913	0.069	0.639	0.115	0.432
	Proteobacteria; Gammaproteobacteria; Burkholderiales; Comamonadaceae; *Aquabacterium*	0.339	**0.017**	0.024	0.868	− 0.030	0.844	0.017	0.914	− 0.132	0.365	0.083	0.570	0.105	0.473	0.307	**0.032**	0.053	0.718	0.285	**0.047**

*ASVs* amplicon sequence variant, *BMI* body mass index, *SE* sleep efficiency, *SOL* sleep onset latency, *ET* end time, *S corr.* Spearman correlation.

Significant p values are shown in bold.

Other positive correlations with PA include the taxa *Catenibacterium* (p = 0.01), *Lachnospira* (p = 0.017), *Clostridium *sensu stricto* 1* (p = 0.013), *Dialister* (p = 0.017), *Winogradskyella* (p = 0.043), *Epibacterium* (p = 0.041), *SAR86 clade* (p = 0.048), *[Eubacterium] coprostanoligenes group* (p = 0.043), *Clostridia vadinBB60 group* (p = 0.014) and *Clostridia UCG-014* (p = 0.015). Negative correlations of PA were found with the taxa *Lachnospiraceae* (p = 0.011), *Marinifilaceae* (p = 0.045), *DTU089* (p = 0.041), *Alistipes* (p = 0.037), *Butyricimonas* (p = 0.033), and *Negativibacillus* (p = 0.025).

### Correlations between measured variables

**Figure 2:**
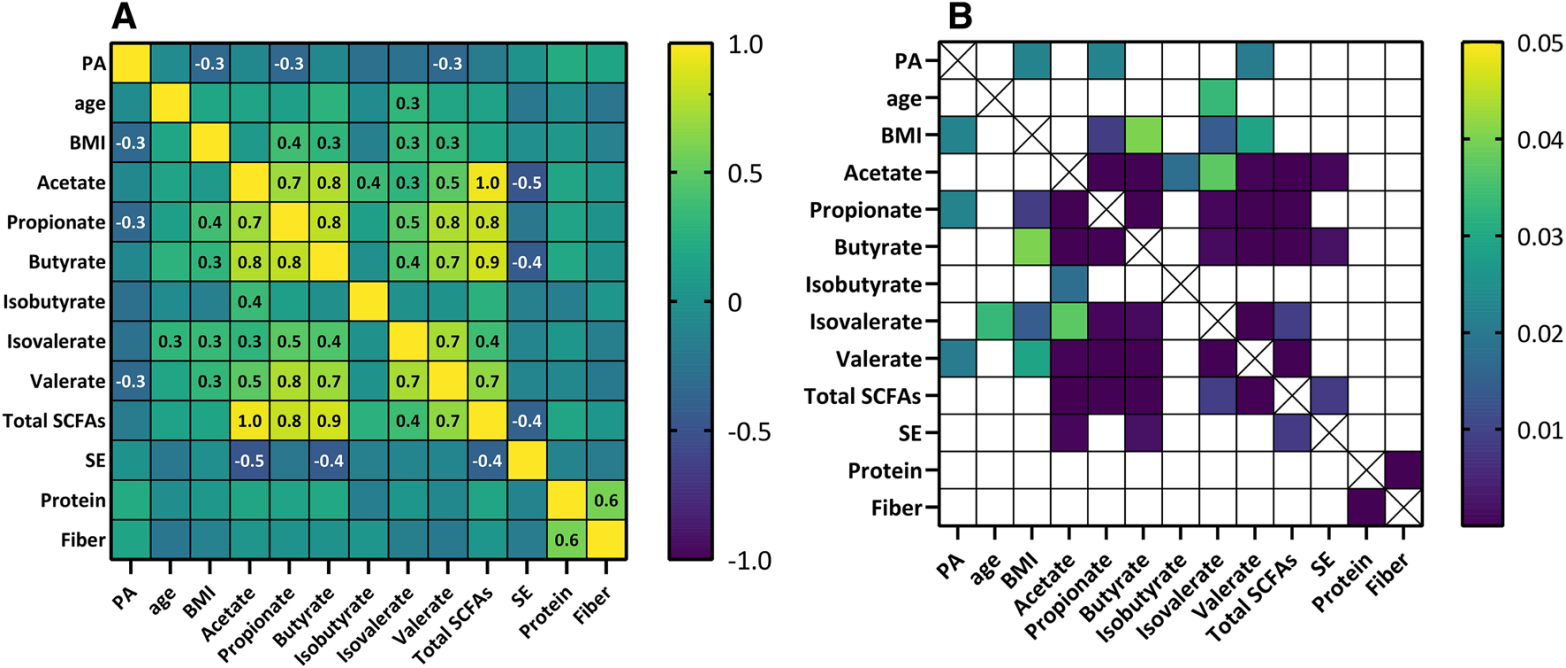
Spearman correlations between measured variables (**A**). All variables are shown in the X and Y-axis. Values of significant correlations are shown in the correspondent squares. P-values of measured variables (**B**). All variables are shown in the X and Y-axis. Color-filled squares represent p-values ≤ 0.05.

Additional correlations were carried out between PA, age, BMI, SCFAs, sleep efficiency, fiber and protein intakes. Correlation results are shown in Fig. 2A and p-value ranges are shown in Fig. 2B. Physical activity was negatively correlated to BMI, propionate, and valerate (p = 0.022, p = 0.022, and p = 0.021, respectively). Age was positively correlated to isovalerate (p = 0.033). SCFAs propionate, butyrate, isovalerate and valerate (p = 0.008, p = 0.040, p = 0.014 and p = 0.029, respectively) correlated positively to BMI. Sleep efficiency was correlated to acetate, butyrate, and total SCFAs (p = 0.001, p = 0.002, and p = 0.009, respectively).

## Discussion

We aimed to determine differences in the gut microbiota composition and its primary metabolites between more active and less active groups of older adults with insomnia and unveil potential connections between specific microbiome taxa and measured variables. The results show different microbiota taxa in each PA group, increased SCFAs in feces of less active individuals, and significant associations between physical activity, gut microbiota, SCFAs, and sleep parameters.

In the current study, the microbial family Erysipelotrichaceae and Peptococcaceae and the genus *Peptococcus* were significantly higher in the more active group (> 6,500 steps/day). Similar results were obtained by studies that evaluated PA by self-reported questionnaires^41–43^. For example, increased Erysipelotrichaceae were shown in individuals that reported frequent exercise^41^ and among adults with higher cardiorespiratory fitness^42^. Furthermore, Erysipelotrichaceae abundances were significantly higher in elite athletes when compared to controls^43^ and in a healthy-aging cohort when compared with non-healthy counterparts^44^. More importantly, these families were also positively associated with higher step counts in community-dwelling older men^12^.

At a genus level, we show positive correlations between *Catenibacterium*, a member of the Erysipelotrichaceae family, and step counts. *Catenibacterium* was also enriched in more active South Korean subjects^45^ and more abundant in top Polish endurance athletes than sedentary controls^46^. In addition, more active individuals were characterized by higher abundances of *Prevotella 9*, *Clostridium *sensu stricto* 1*, and *UCG-002*; the latter two have also been positively correlated to PA. These results are consistent with previous work showing higher levels of these taxa in athletes^46^. For more sedentary groups, the results are contradictory. We show higher abundances of *Lachnoclostridium, Blautia, Barnesiella*, and *UCG-005* in less active individuals, opposing previous publications^46–48^.

Several mechanisms have been proposed to explain the interrelationship between physical activity and microbiota composition, such as antioxidant activity, immune modulation, gastrointestinal permeability, and the production of metabolites such as SCFAs^49^. Although SCFAs can influence a wide array of host systems and metabolic pathways^50,51^, the mechanism by which PA modulate SCFAs and vice-versa remains speculative^51^. Furthermore, associations between PA and SCFAs have not been extensively studied. Existing publications focused mainly on the effects of exercise interventions on healthy young adults, and the results shown are inconsistent^14,42,52,53^. In the present study, more active individuals had significantly lower levels of fecal SCFAs despite similar demographics, health characteristics, sleep parameters, and recorded fiber and protein intake between groups. Correlation analysis showed negative associations of step-counts with SCFAs propionate and valerate. Surprisingly, *[Ruminococcus] gauvreauii group*, a member of the Lachnospiraceae family and a SCFAs producer^54^, was abundant in the more active group but correlated negatively to propionate and valerate. Other gut microbiota members such as the genus *Monoglobus and Ruminococcus* correlated negatively to PA and were positively associated with isobutyrate and isovalerate levels, respectively.

Although all participants were older adults with insomnia, we examined the associations between sleep parameters, SCFAs, microbiota, and PA. SE was negatively associated with SCFAs (acetate and butyrate) and total SCFAs. Genus *Monoglobus*, associated with PA and isobutyrate, was positively associated with SE and negatively associated with SOL. These results may imply the existence of a link between physical activity, gut microbiota, SCFAs, and sleep. To the best of our knowledge, this is the first study to show associations between gut microbiota, SCFA concentrations, and sleep quality in older individuals.

Other important results include positive associations between BMI and SCFAs butyrate, propionate, isovalerate and valerate. BMI was inversely correlated with PA and with the genera *[Ruminococcus] gauvreauii group* and *Holdemanella*, both abundant in the more active group. The relationship between BMI gut microbiota and SCFAs produced by intestinal bacteria is not yet fully understood^55^, but SCFAs represent an important energy source for the human body, contributing to lipogenesis and accumulation in adipocytes, leading to energy harvest^56^. Moreover, higher fecal SCFA concentrations may be associated with gut dysbiosis, gut permeability, excess adiposity, and cardiometabolic risk factors^57^.

This study has some limitations. The sample is relatively small and homogenous; however, since this is the first study to explore community-dwelling older adults with insomnia, the similarity between the groups enables us to extract the effect of physical activity with fewer biases. Indeed, physical activity can be quantified in many ways and step count is not the sole and or necessarily the ultimate representation of PA; however, this consistent and objective measure gains greater validity due to its accuracy and relevance to older adults’ daily life. Additionally, since there is no agreed-upon recommendation for daily step count for older adults, we used large population-based studies^11^ to support the cut-off we used; future studies should explore a variety of doses for various sub-groups with different health characteristics.

## Conclusion

In conclusion, physical activity is undoubtedly associated with positive outcomes. However, the role of the gut microbiota composition and its metabolites as modulators is still poorly understood and requires further research. Our findings add to the existing literature in elucidating the mechanism underlying these relationships in older adult populations with insomnia.

## Supplementary Information

Supplementary Information.

## Data Availability

The datasets generated and/or analysed during the current study are available in the NCBI-SRA repository: https://dataview.ncbi.nlm.nih.gov/object/PRJNA730721?reviewer=tn09eubuh30u1rqjujd21o8454. The raw SCFA data and actigraph measures for each participant and which was used for the analysis are available from the corresponding author upon request.

## References

[1] Bloom HG (2009). Evidence-based recommendations for the assessment and management of sleep disorders in older persons: Supplement. J. Am. Geriatr. Soc..

[2] American Psychiatric Association. *American Psychiatric Association, 2013. Diagnostic and statistical manual of mental disorders (5th ed.)*. *American Journal of Psychiatry* (2013). 10.1176/appi.books.9780890425596.744053.

[3] Ancoli-Israel, S. & Shochat, T. Insomnia in Older Adults. in *Principles and Practice of Sleep Medicine* 1544–1550 (Elsevier, 2011). 10.1016/B978-1-4160-6645-3.00135-3.

[4] Fernandez-Mendoza J (2017). Insomnia symptoms with objective short sleep duration are associated with systemic inflammation in adolescents. Brain. Behav. Immun..

[5] Fernandez-Mendoza J (2010). Insomnia with objective short sleep duration is associated with deficits in neuropsychological performance: A general population study. Sleep.

[6] Ba D, Ev N, Dm B, Jl M, Cb C (2017). Interrelationship between sleep and exercise: A systematic review. Adv. Prev. Med..

[7] Reid KJ (2010). Aerobic exercise improves self-reported sleep and quality of life in older adults with insomnia. Sleep Med..

[8] Baron KG, Reid KJ, Zee PC (2013). Exercise to improve sleep in insomnia: Exploration of the bidirectional effects. J. Clin. Sleep Med..

[9] Buman MP, Hekler EB, Bliwise DL, King AC (2011). Exercise effects on night-to-night fluctuations in self-rated sleep among older adults with sleep complaints. J. Sleep Res..

[10] Kraus WE (2019). Daily step counts for measuring physical activity exposure and its relation to health. Med. Sci. Sports Exerc..

[11] Tudor-Locke C (2011). How many steps/day are enough? For older adults and special populations. Int. J. Behav. Nutr. Phys. Act..

[12] Langsetmo L (2019). The association between objectively measured physical activity and the gut microbiome among older community dwelling men. J. Nutr. Heal. Aging.

[13] Oftedal S (2020). Daily steps and diet, but not sleep, are related to mortality in older Australians. J. Sci. Med. Sport.

[14] Tzemah R (2020). Attributes of physical activity and gut microbiome in adults: A systematic review. Int. J. Sports Med..

[15] Ortiz-Alvarez L, Xu H, Martinez-Tellez B (2020). Influence of exercise on the human gut microbiota of healthy adults: A systematic review. Clin. Transl. Gastroenterol..

[16] Mitchell CM (2019). Does exercise alter gut microbial composition? A systematic review. Med. Sci. Sports Exerc..

[17] Mach N, Fuster-Botella D (2017). Endurance exercise and gut microbiota: A review. J. Sport Heal. Sci..

[18] Morita E (2019). Aerobic exercise training with brisk walking increases intestinal bacteroides in healthy elderly women. Nutrients.

[19] Zhu Q, Jiang S, Du G (2020). Effects of exercise frequency on the gut microbiota in elderly individuals. Microbiologyopen.

[20] Taniguchi H (2018). Effects of short-term endurance exercise on gut microbiota in elderly men. Physiol. Rep..

[21] Inoue T (2018). Effect of combined bifidobacteria supplementation and resistance training on cognitive function, body composition and bowel habits of healthy elderly subjects. Benef. Microbes.

[22] Vancamelbeke M, Vermeire S (2017). The intestinal barrier: A fundamental role in health and disease. Expert Rev. Gastroenterol. Hepatol..

[23] Makki K, Deehan EC, Walter J, Bäckhed F (2018). The impact of dietary fiber on gut microbiota in host health and disease. Cell Host Microbe.

[24] Evans JM, Morris LS, Marchesi JR (2013). The gut microbiome: The role of a virtual organ in the endocrinology of the host. J. Endocrinol..

[25] de la Cuesta-Zuluaga J (2018). Higher fecal short-chain fatty acid levels are associated with gut microbiome dysbiosis, obesity, hypertension and cardiometabolic disease risk factors. Nutrients.

[26] Vogt JA, Wolever TMS (2003). Fecal acetate is inversely related to acetate absorption from the human rectum and distal colon. J. Nutr..

[27] Szentirmai É, Millican NS, Massie AR, Kapás L (2019). Butyrate, a metabolite of intestinal bacteria, enhances sleep. Sci. Rep..

[28] Heath ALM (2020). Association between the faecal short-chain fatty acid propionate and infant sleep. Eur. J. Clin. Nutr..

[29] Zhang X (2021). Age-related compositional changes and correlations of gut microbiome, serum metabolome, and immune factor in rats. GeroScience.

[30] Magzal F (2021). Associations between fecal short-chain fatty acids and sleep continuity in older adults with insomnia symptoms. Sci. Rep..

[31] Vaccaro A (2020). Sleep loss can cause death through accumulation of reactive oxygen species in the gut. Cell.

[32] UN. *World Population Ageing 2019*. *World Population Ageing 2019* (2019).

[33] Kukull WA, Larson EB, Teri L, Bowen J, McCormick W, Pfanschmidt ML (1994). The Mini-Mental State Examination score and the clinical diagnosis of dementia. J. Clin. Epidemiol..

[34] Faul F, Erdfelder E, Lang AG, Buchner A (2007). G*Power 3: A flexible statistical power analysis program for the social, behavioral, and biomedical sciences. Behav. Res. Methods.

[35] Sasaki JE, John D, Freedson PS (2011). Validation and comparison of ActiGraph activity monitors. J. Sci. Med. Sport.

[36] Shahar D, Fraser D, Shai I, Vardi H (2003). Development of a food frequency questionnaire (FFQ) for an elderly population based on a population survey. J. Nutr..

[37] Caporaso JG (2012). Ultra-high-throughput microbial community analysis on the Illumina HiSeq and MiSeq platforms. ISME J..

[38] Rstudio, T. RStudio: Integrated Development for R. *Rstudio Team, PBC, Boston, MA.* http://www.rstudio.com/ (2020). 10.1145/3132847.3132886.

[39] Chong J, Liu P, Zhou G, Xia J (2020). Using MicrobiomeAnalyst for comprehensive statistical, functional, and meta-analysis of microbiome data. Nat. Protoc..

[40] Silva. https://www.arb-silva.de/.

[41] Mcfadzean R (2014). Exercise can help modulate human gut microbiota. Undergrad. Honor. Theses..

[42] Estaki M (2016). Cardiorespiratory fitness as a predictor of intestinal microbial diversity and distinct metagenomic functions. Microbiome.

[43] Clarke SF (2014). Exercise and associated dietary extremes impact on gut microbial diversity. Gut.

[44] Singh H (2019). Gastro-intestinal and oral microbiome signatures associated with healthy aging. GeroScience.

[45] Shin J-H, Sim M, Lee J-Y, Shin D-M (2016). Lifestyle and geographic insights into the distinct gut microbiota in elderly women from two different geographic locations. J. Physiol. Anthropol..

[46] Kulecka M (2020). The composition and richness of the gut microbiota differentiate the top Polish endurance athletes from sedentary controls. Gut Microbes.

[47] Tabone M (2021). The effect of acute moderate-intensity exercise on the serum and fecal metabolomes and the gut microbiota of cross-country endurance athletes. Sci. Rep..

[48] Muralidharan J (2021). Effect on gut microbiota of a 1-y lifestyle intervention with Mediterranean diet compared with energy-reduced Mediterranean diet and physical activity promotion: PREDIMED-Plus Study. Am. J. Clin. Nutr..

[49] Hughes RL (2020). A review of the role of the gut microbiome in personalized sports nutrition. Front. Nutr..

[50] Silva YP, Bernardi A, Frozza RL (2020). The role of short-chain fatty acids from gut microbiota in gut-brain communication. Front. Endocrinol..

[51] Carey RA, Montag D (2021). Exploring the relationship between gut microbiota and exercise: short-chain fatty acids and their role in metabolism. BMJ Open Sport Exerc. Med..

[52] Allen JM (2018). Exercise Alters Gut Microbiota Composition and Function in Lean and Obese Humans. Med. Sci. Sports Exerc..

[53] Barton W (2018). The microbiome of professional athletes differs from that of more sedentary subjects in composition and particularly at the functional metabolic level. Gut.

[54] Vacca M (2020). The controversial role of human gut lachnospiraceae. Microorganisms.

[55] Kim KN, Yao Y, Ju SY (2019). Short chain fatty acids and fecal microbiota abundance in humans with obesity: A systematic review and meta-analysis. Nutrients.

[56] M, D., EE, B. & WM, de V. Do nutrient-gut-microbiota interactions play a role in human obesity, insulin resistance and type 2 diabetes? *Obes. Rev.* **12**, 272–281 (2011). 10.1111/j.1467-789X.2010.00797.x 20804522

[57] de la Cuesta-Zuluaga J (2019). Higher fecal short-chain fatty acid levels are associated with gut microbiome dysbiosis, obesity, hypertension and cardiometabolic disease risk factors. Nutrients.

